# Predictive value of lymphocyte to monocyte ratio and monocyte to high-density lipoprotein ratio for acute deep vein thrombosis after total joint arthroplasty: a retrospective study

**DOI:** 10.1186/s13018-018-0910-2

**Published:** 2018-08-24

**Authors:** Xiaobo Zhu, Yao Yao, Chen Yao, Qing Jiang

**Affiliations:** 1 Department of Sports Medicine and Adult Reconstructive Surgery, Nanjing Drum Tower Hospital, Clinical College of Nanjing Medical University, Nanjing, China; 2 Department of Sports Medicine and Adult Reconstructive Surgery, Nanjing Drum Tower Hospital Affiliated with the Medical School of Nanjing University, Nanjing, 210008 Jiangsu China

**Keywords:** Deep vein thrombosis, Lymphocyte to monocyte ratio, Monocyte to HDL ratio, Total joint arthroplasty

## Abstract

**Background:**

Deep vein thrombosis (DVT) is one of the most dangerous complications of total joint arthroplasty (TJA). Systemic inflammation has proved to have a great contribution to thrombosis and has been considered as a risk factor for DVT recently. The lymphocyte to monocyte ratio (LMR) and monocyte to high-density lipoprotein (HDL) ratio (MHR) are two biomarkers used widely for systemic inflammation. This study aims to find out the potential predictive value of LMR and MHR for DVT after TJA.

**Methods:**

A total of 853 patients who underwent primary TJA were finally included in this retrospective study. Acute DVT after TJA was evaluated by venography. Preoperative and postoperative LMR and MHR were calculated according to the blood routine test and blood biochemistry test. The association between LMR or MHR and DVT and their predictive value were evaluated by multiple logistic regression analysis and ROC curve respectively.

**Results:**

Totally, 126 patients (14.8%) were diagnosed with DVT by venography. Patients with DVT had a significantly higher level of preoperative MHR (*P* < 0.001) and postoperative MHR (*P* < 0.001), along with a significantly lower level of preoperative LMR (*P* < 0.001) and postoperative LMR (*P* < 0.001). Multiple logistic regression indicated that BMI (OR = 1.10, *P* = 0.001), preoperative LMR (OR = 0.72, P<0.001), and postoperative LMR (OR = 0.32, *P* < 0.001) were independent risk factors for DVT. Besides, BMI (OR = 1.17, *P* = 0.001), female (OR = 4.6, *P* = 0.004), preoperative MHR (OR = 10.43, *P* = 0.008), postoperative Hb (OR = 0.96, *P* = 0.002), and postoperative LMR were independently associated with symptomatic DVT. The ROC curve suggested that the postoperative LMR had a potential to predict DVT after TJA.

**Conclusion:**

In summary, the present study found out a significant association of perioperative LMR or MHR with DVT after TJA. Moreover, the postoperative LMR had a potential to predict DVT accurately.

## Background

Total joint arthroplasty (TJA) is an effective surgery that helps reestablish the normal axis of the lower limb and improve the quality of life and joint function of patients affected by osteoarthritis or other arthropathies [1, 2]. TJA is a mature surgery with a high satisfaction rate and a low rate of complication [3]. However, deep vein thrombosis (DVT), one of the major complications after TJA, contributes to some severe consequences like pulmonary embolism (PE), which may occur suddenly and cause death quickly [4]. Although there exists a great deal of medical or physical prophylaxis for DVT, it still leads to a high healthy cost, morbidity, and mortality. Unfortunately, DVT cannot be precisely predicted due to the complexity of its diverse risk factors and lack of subject symptoms and clinical signs. Hence, the accurate predictive marker for DVT is quite in urgent need. Previous studies have concentrated on serology, trying to discover the potential biomarkers like D-dimer or P-selection to detect DVT, but the accuracy is not so satisfying [5].

Recently, increasing researchers has viewed inflammation as a vital risk for DVT [6]. Inflammation can cause release of various proinflammatory and prooxidant cytokines from leukocytes, which promoted coagulation and inhibited fibrinolysis [7]. Moreover, excessive proinflammatory and prooxidant cytokines also do harm to blood vessel walls, making the condition worse [8]. Considering the crucial role inflammation plays in initiation and progression of thrombosis, various inflammatory biomarkers including CRP (C-reactive protein) and IL-6 (interleukin-6) have been reported to be valuable to predict DVT [6, 8, 9].

In addition, several parameters obtained from complete blood count, like the neutrophil to lymphocyte ratio (NLR) and the platelet to lymphocyte ratio (PLR), are found to be potential markers of systemic inflammation that have been shown to have prognostic value in cardiovascular and oncological diseases [10, 11]. However, these two parameters have also proved to have poor prognostic value in predicting DVT after TJA [12]. Recently, the lymphocyte to monocyte ratio (LMR) and monocyte to high-density lipoprotein (HDL) ratio (MHR) are defined as another two biomarkers in predicting thrombosis caused by systemic inflammation in cardiovascular and oncological events [13–15]. Meanwhile, the relationship between the plasma LMR or MHR level and the acute DVT after TJA has not been illustrated yet, which pushed us to grope for the predictive or diagnostic value of LMR and MHR.

Our study was conducted to investigate the predictive or diagnostic value of LMR and MHR for DVT after TJA.

## Methods

### Participants and measurements

We conducted a retrospective analysis of the patients consecutively admitted for primary TKA or THA in Nanjing Drum Tower hospital affiliated to Medical School of Nanjing University. The period of recruitment time varied from October 2010 to December 2014. We included the patients who were diagnosed with hip or knee osteoarthritis, rheumatoid arthritis, fracture of femoral neck, avascular necrosis of femoral head, and so on. Meanwhile, we excluded the patients with infectious diseases or clinical signs of infection, neoplasia, inflammatory disease (rheumatic arthritis and Crohn diseases), hematological disorders, serious renal dysfunction, hepatic dysfunction, and current use of immunosuppressive agents as well as with incomplete information. This study was approved by the hospital ethics review committee.

All surgeries were performed by three experienced surgeons. All patients received conventional thromboembolism prophylaxis therapy (low-molecular-weight heparin) and underwent the same rehabilitation program after surgery. Low-molecular-weight heparin was injected subcutaneously at a dose of 30 mg once a day.

Routine venography of the affected lower limb was performed during 3–5 days after operation. DVT was diagnosed by three experienced radiologists according to Robinov group’s criterion [16]. Symptomatic DVT was characterized by the symptoms of pain and swelling of the leg, skin discoloration, and a positive Homans’ sign [17] or Neuhof’s sign [18] within 30 days after surgery. Proximal DVT was defined as thrombosis at the level of popliteal vein or above while distal DVT was defined as thrombosis occurring within the calf veins.

The blood specimen was collected from the peripheral venous before the operation and on the first morning after the operation. The blood routine test was performed in the clinical laboratory of Nanjing Drum Tower Hospital affiliated to Medical School of Nanjing University. Preoperative and postoperative LMR and MHR were calculated according to routine blood tests and blood biochemistry tests. In addition, the perioperative D-dimer test and patients’ basic demographic and clinical characteristics (age, gender, BMI, hypertension, insulin resistance, smoking history, heart diseases, malignancy, and thrombosis history) were also recorded.

### Statistical analysis

Statistical analysis was performed using SPSS 17.0 system software (SPSS Inc., Chicago, IL, USA). Numeric data were shown as mean ± standard deviation. Categorical data were shown as numbers with percentages. Categorical variables including gender, hypertension, insulin resistance, smoking history, malignancy, heart disease, and thrombosis history were compared by a chi-square test. Continuous variables including age, BMI, LMR, and MHR were analyzed by the Student *t*-test. The association of LMR or MHR with total DVT or symptomatic DVT was investigated using multiple logistic regression after adjustment for other variables. The OR (odd ratio) and 95% CIs (confidence intervals) were calculated for each associated variable. ROC (receiver-operating characteristic) curve was performed by MedCalc 11.5 (MedicReS, New York, NY) to identify the sensitivity and specificity of postoperative Hb (hemoglobin) and perioperative LMR and MHR for predictive value of DVT. *P* < 0.05 was considered significant in all statistical analysis.

## Results

Finally, a total of 853 patients containing 273 TKA (total knee arthroplasty) and 580 THA (total hip arthroplasty) were included in our study. There were 608 female patients and 245 male patients in this study. The mean age of patients was 64.0 ± 12.9 years (range 18 to 93 years). One hundred and twenty-six patients (14.8%) were diagnosed with DVT, including 111 (13.0%) with distal DVT and 15 (1.8%) with proximal DVT. In addition, 58 patients (6.7%) developed symptomatic DVT and 795 patients (93.2%) developed a non-symptomatic DVT. There was no fatal pulmonary embolism (PE) in this study. Demographic characteristics and clinical characteristics of patients are summarized in Table 1. Patients with DVT had a greater mean baseline of age (*P* = 0.001) and malignant history (*P* = 0.009). Moreover, patients with symptomatic DVT showed a greater mean baseline of female gender (*P* = 0.021) and BMI (*P* < 0.001).

**Table 1:** Characteristic data of subjects

Variables	Total patients			Total patients		
	Without DVT (*n* = 727)	With DVT (*n* = 126)	*P* value	Without symptom (*n* = 795)	With symptom (*n* = 58)	*P* value
Age (years)	63.5 ± 13.8	67.7 ± 10.3	0.001	64.0 ± 12.9	65.6 ± 12.2	0.358
Gender (female)	509(70%)	99(78.6%)	0.05	559(70.3%)	49(84.5%)	0.021
BMI	24.5 ± 4.4	25.5 ± 4.5	0.018	24.6 ± 4.4	26.8 ± 3.7	< 0.001
Hypertension	282(38.8%)	56(44.4%)	0.231	313(39.4%)	25(43.1%)	0.575
Insulin resistance	96(13.2%)	17(13.5%)	0.93	102(12.8%)	11(19.0%)	0.183
Smoking history	69(9.5%)	6(4.8%)	0.084	72(9.1%)	4(6.9%)	0.577
Heart disease	64(8.8%)	8(6.3%)	0.36	66(8.3%)	6(10.3%)	0.589
Malignancy	26(2.2%)	8(6.3%)	0.009	29(3.6%)	5(8.6%)	0.062
Thrombosis history	75(10.3%)	10(7.9%)	0.408	79(9.9%)	6(10.3%)	0.920

*P* < 0.05 was considered statistically significant

The differences of perioperative laboratory examinations between groups with or without DVT are compared in Table 2. Patients with DVT showed a significant higher level of postoperative MHR (*P* < 0.001) in comparison with those without DVT. In contrast, the level of preoperative LMR (*P* < 0.001), postoperative LMR (*P* < 0.001), and postoperative fibrinogen (*P* = 0.001) were lower in DVT group. The same tendency was seen when comparing group with symptomatic DVT to that without symptomatic DVT. Preoperative LMR, postoperative LMR, and postoperative fibrinogen are lower in group with symptomatic DVT while postoperative MHR was higher in symptomatic DVT group. In addition, the group with symptomatic DVT also showed higher preoperative MHR in comparison to that without symptomatic DVT. There was no significant difference in other parameters between these two groups. When we focus on the DVT position by dividing them into proximal DVT group and distal DVT group according to the thrombosis position, as shown in Table 3, we found that the levels of preoperative MHR (*P* = 0.01) and postoperative MHR (*P* < 0.001) in the group with proximal DVT were significantly higher than that with distal DVT. Besides, the level of preoperative LMR (*P* < 0.001) was significantly lower in proximal DVT group than that in distal DVT group.

**Table 2:** Perioperative laboratory data of patients

Variables	Total patients			Total patients		
	Without DVT (*n* = 727)	With DVT (*n* = 126)	*P* value	Without symptomatic DVT (*n* = 795)	With symptomatic DVT (*n* = 58)	*P* value
Preoperative data						
RBC(× 10^12^/L)	4.3 ± 0.5	4.2 ± 0.5	0.402	4.3 ± 0.5	4.2 ± 0.5	0.426
Hb (g/L)	127.8 ± 15.3	128.2 ± 14.8	0.79	128.0 ± 15.2	124.5 ± 13.8	0.080
WBC(× 10^9^/L)	6.0 ± 1.7	6.0 ± 2.1	0.825	6.0 ± 1.8	5.9 ± 1.5	0.78
D-dimmer (mg/L)	1.5 ± 3.5	1.5 ± 2.9	0.988	1.5 ± 3.4	1.5 ± 2.2	0.97
Fibrinogen (g/L)	3.3 ± 0.8	3.3 ± 0.8	0.562	3.4 ± 0.8	3.2 ± 0.8	0.19
LMR	5.9 ± 4.5	2.7 ± 1.8	< 0.001	5.9 ± 4.8	2.8 ± 1.8	< 0.001
MHR	0.3 ± 0.2	0.4 ± 0.4	< 0.001	0.3 ± 0.2	0.4 ± 0.3	< 0.001
Postoperative data						
RBC(×10^12^)	3.6 ± 0.6	3.7 ± 1.0	0.072	3.7 ± 0.7	3.6 ± 1.1	0.597
Hb (g/L)	108.0 ± 16.1	110.9 ± 14.7	0.055	108.8 ± 16.1	103.9 ± 14.1	0.023
WBC(×10^9^/L)	10.8 ± 3.4	10.5 ± 3.3	0.43	10.7 ± 3.4	11.1 ± 3.6	0.402
D-dimmer (mg/L)	2.9 ± 3.7	3.4 ± 5.8	0.159	2.2 ± 2.4	2.8 ± 2.0	0.063
Fibrinogen (g/L)	4.6 ± 0.98	4.3 ± 0.9	< 0.001	4.6 ± 1.2	4.2 ± 0.8	0.032
LMR	4.2 ± 2.8	1.5 ± 2.2	< 0.001	2.2 ± 1.0	1.3 ± 0.9	< 0.001
MHR	0.4 ± 0.2	0.6 ± 0.5	< 0.001	0.4 ± 0.26	0.8 ± 0.5	< 0.001

*P* < 0.05 was considered statistically significant

**Table 3:** Distribution of perioperative LMR and MHR between distal DVT and proximal DVT

Variables	Distal DVT (*n* = 111)	Proximal DVT (*n* = 15)	*P* value
Preoperative			
LMR	5.8 ± 6.3	2.9 ± 1.9	0.09
MHR	0.3 ± 0.2	0.4 ± 0.2	0.01
Postoperative			
LMR	4.7 ± 3.5	1.1 ± 0.7	< 0.001
MHR	0.3 ± 0.2	0.7 ± 0.3	< 0.001

*P* < 0.05 was considered statistically significant

We used logistic regression to reveal the relationship between the underlying risk factors and DVT. The results of multiple logistic regression in Table 4 indicated that BMI (OR = 1.10, *P* = 0.001), preoperative LMR (OR = 0.72, *P* < 0.001), and postoperative LMR (OR = 0.32, *P* < 0.001) were independently associated with DVT. Moreover, BMI (OR = 1.17, *P* = 0.001), female gender (OR = 4.6, *P* = 0.004), preoperative MHR (OR = 10.43, *P* = 0.008), postoperative LMR (OR = 0.12, *P* < 0.001), and postoperative Hb (OR = 0.96, *P* = 0.002) were independent risk factors for symptomatic DVT, which was also shown in Table 4.

**Table 4:** Multiple logistic regression analyses for predictors of DVT or symptomatic DVT after adjustment

Variables	Total DVT events		Variables	Symptomatic DVT events	
	OR(95%CI)	*P* value		OR(95%CI)	*P* value
Age	1.00(0.983~1.023)	0.777	BMI	1.17(1.07~1.29)	0.001
BMI	1.10(1.039~1.158)	0.001	Gender (Female)	1.00(0.983~1.023)	0.004
Malignancy	1.93(0.554~6.691)	0.303	LMR (pre-op)	1.10(1.039~1.158)	0.499
LMR (pre-op)	0.72(0.616~0.844)	< 0.001	MHR (pre-op)	1.93(0.554~6.691)	0.008
MHR (pre-op)	0.72(0.274~1.895)	0.506	Hb (post-op)	0.72(0.616~0.844)	0.002
Fibrinogen (post-op)	0.87(0.651~1.169)	0.36	Fibrinogen (post-op)	0.72(0.274~1.895)	0.818
LMR (post-op)	0.32(0.236~0.424)	< 0.001	LMR (post-op)	0.87(0.651~1.169)	< 0.001
MHR (post-op)	1.63(0.636~4.186)	0.309	MHR (post-op)	0.32(0.236~0.424)	0.559

*P* < 0.05 was considered statistically significant

**Fig. 1:**
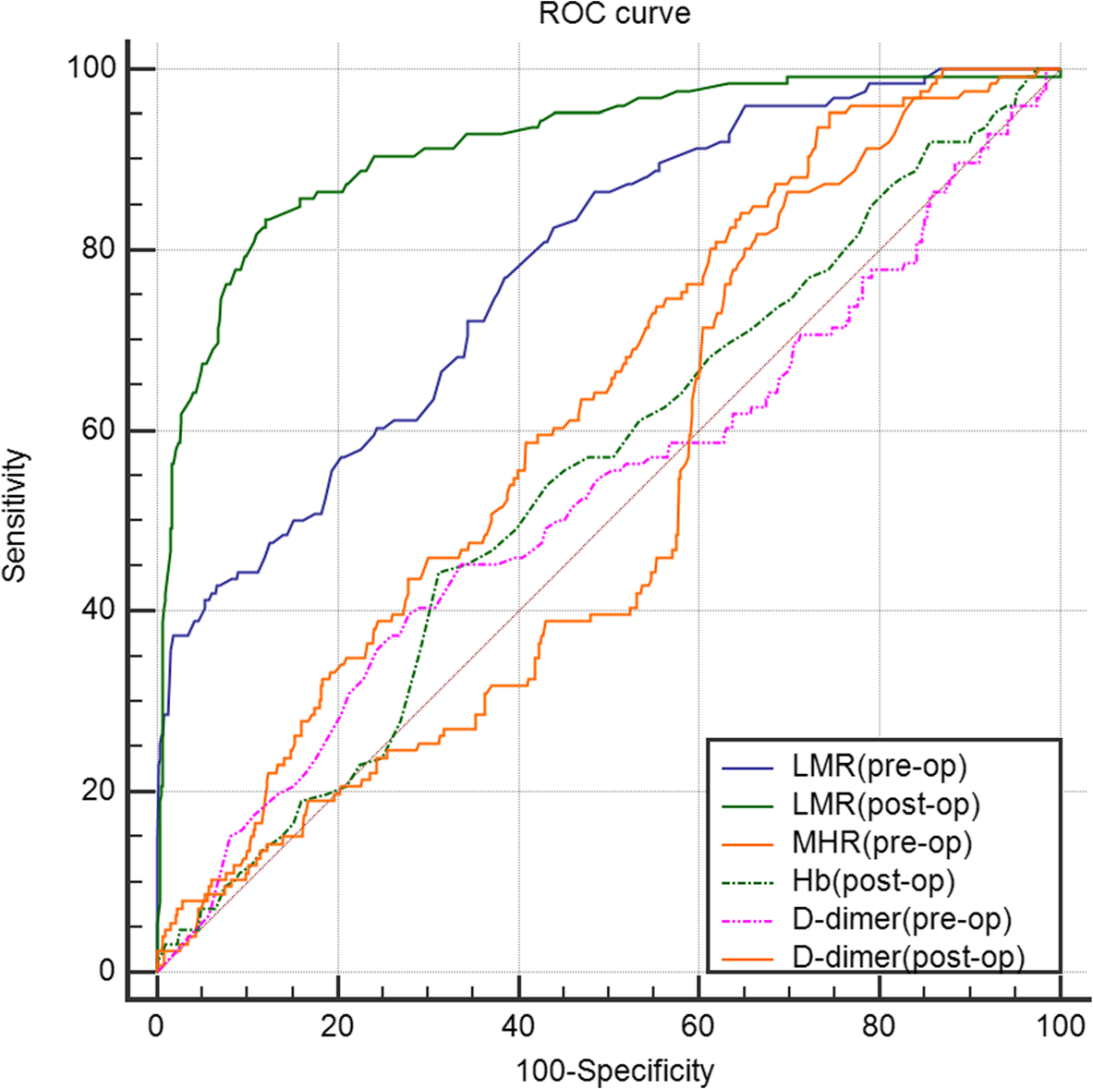
ROC curve of D-dimer, Hb, LMR, and MHR to predict DVT. The ROC curve analysis and AUC demonstrated the specificity and sensitivity of perioperative D-dimer, preoperative LMR, and postoperative Hb in predicting DVT were low. But the postoperative LMR had a high sensitivity and specificity to predict DVT

As shown in Fig. 1 and Table 5, the ROC curve and AUC (areas under the curve) indicated the specificity and sensitivity of postoperative Hb, preoperative LMR, and perioperative D-dimer is very low. However, the AUC of postoperative LMR was 0.92 (95% CI 0.90~0.93), along with a specificity of 87.9% and a sensitivity of 83.3%, which implied that the postoperative LMR had a high predictive value for DVT. Besides, the AUC of preoperative MHR had a low AUC but a high sensitivity up to 95.2%. Considering the severe outcome of DVT, the MHR might have a receivable potential to detect DVT due to the great sensitivity of the test.

**Table 5:** AUC of the ROC curve and 95% confidence interval of preoperative LMR, postoperative LMR, preoperative MHR, postoperative Hb, and perioperative D-dimer

Variables	AUC	95%CI	*P* value	Sensitivity (%)	Specificity (%)
Hb (post-op)	0.55	0.52~0.58	0.073	44.4	68.8
LMR (pre-op)	0.78	0.75~0.81	< 0.001	82.5	56.1
MHR (pre-op)	0.63	0.59~0.66	< 0.001	95.2	25.5
LMR (post-op)	0.92	0.90~0.93	< 0.001	83.3	87.9
D-dimer (pre-op)	0.53	0.49~0.56	0.35	39.7	72.2
D-dimer (post-op)	0.52	0.49~0.55	0.42	86.5	30.1

*P* < 0.05 was considered statistically significant

## Discussion

To our knowledge, this is the first study aiming to identify the association between LMR or MHR and acute DVT after TJA. We emphasized that the low level of preoperative and postoperative LMR and high level of preoperative and postoperative MHR was significantly associated with DVT after primary TJA. Furthermore, the ROC curve analysis illustrated that the postoperative LMR had a high predictive value for DVT while preoperative MHR had a receivable potential to detect DVT after primary TJA due to a great sensitivity.

As is known, surgery-induced inflammation plays an important role in DVT after surgery for constructing a pro-coagulate microenvironment [19]. Previous studies pointed out a higher level of leukocyte after neurosurgery and cardiac surgery [20, 21]. Meanwhile, another study also found out a higher level of inflammatory parameter like leukocyte count and CRP after orthopedic surgery and some of the inflammatory biomarkers were independently associated with DVT [22]. For instance, Yombi et al. [23] compared NLR with CRP to evaluate the better one to predict DVT after TJA. They found that NLR distribution showed a better kinetic pattern than CRP distribution for the follow-up of early inflammation after total knee arthroplasty. Also, Barker et al. [24] reported that the parameter NLR was correlated with systemic inflammation and the increase of NLR indicated a higher patient susceptibility of sustaining VTE after TJA, but the shortcoming of their study was also obvious that the patient number was just 20. Studies in our institution also identified the significant association between the systemic inflammatory parameter perioperative NLR or PLR and acute DVT after TJA, but the accuracy of their predictive value was not so satisfying [12]. Given the easy access and low expense of routine blood test and blood biochemistry test, we tried to make full use of routine blood test and blood biochemistry to discover other potential biomarkers to predict DVT conveniently.

Recently, increasing researchers used LMR and MHR to predict systemic inflammation and thrombosis in coronary artery diseases and oncological diseases [25, 26]. However, studies concerning LMR or MHR as a biomarker for predicting acute DVT after TJA were not available yet. Fortunately, we found that preoperative and postoperative LMR were significantly lower in the patients with DVT, especially symptomatic DVT. Besides, the preoperative MHR was significantly higher in patients with DVT, which implied that a relatively higher level of monocytes and a relatively lower level of HDL and lymphocytes. The variation tendency was consistent with the former research [27]. The mechanism explaining variation tendency remained controversial. As we all know, monocytes play a vital role in the initiation and progression of inflammation [28]. When activated monocytes interact with damaged endothelium, they secrete proinflammatory cytokines and adhesion molecules, leading to thrombosis. Additionally, many studies had pointed out that the imbalance between lymphocytes and monocytes aggravated the inflammation and thrombosis trend. On the contrary, HDL functions in the opposite way. Various studies have shown that HDL improves endothelial function via its anti-inflammatory and antioxidative effects. Last but not least, HDL also attenuates thrombosis by modulating monocyte activation, adhesion, and transmigration [29, 30].

Furthermore, apart from comparing the LMR or MHR between the group with DVT and without DVT, we also compared the LMR and MHR between group with proximal DVT and distal DVT according to the position of DVT. We concluded that the position of DVT also made a difference. Our study suggested that the group with proximal DVT presented a significantly lower level of postoperative LMR and a significantly higher level of perioperative MHR than that with distal DVT, which implied that patients with proximal DVT might have a worse inflammatory status and should be paid more attention to prevent the further severe clinical outcomes. Some study also had demonstrated that the level of inflammatory parameters was higher in patients with proximal DVT than those with distal DVT [22]. Their research confirmed our findings indirectly. Furthermore, the ROC curve analysis was calculated to evaluate the predictive value of perioperative LMR and MHR. According to the AUC, the sensitivity and specificity suggested that postoperative LMR had a reasonable value for predicting DVT, better than preoperative and postoperative D-dimer, a conventional parameter used to predict DVT. Meanwhile, the AUC of preoperative LMR is low and subsequently limited its predictive value for DVT. Additionally, given the severe outcomes the DVT could bring out, the miss diagnosis of DVT would lead to a fatal outcome. That is to say, the sensitivity is relatively more important in the diagnostic test. As a result, we were surprised to find that the sensitivity of preoperative MHR was high, along with a low specificity, which meant that patients susceptible to suffer from DVT can be easily screened out by preoperative MHR. So, further research might be conducted to find out the embedded association between MHR and DVT.

To be honest, our study has several limitations. First, our study is a retrospective study. Second, we performed venography just in the operated lower limbs, ignoring the possibility of DVT on the non-operated lower limb. Third, the surgery was operated by three different surgeons, which would result in different possibility of DVT. Last but not least, we only tested for early postoperative DVT, which may lead to missing cases with DVT later down the track.

## Conclusion

In conclusion, the present study emphasized the significant association of perioperative LMR and MHR with acute DVT after TJA. Moreover, the postoperative LMR had a potential to predict DVT after TJA accurately.

## Abbreviations

ACU: Areas under the curve
BMI: Body mass index
CIs: Confidence intervals
CRP: C-reactive protein
DVT: Deep vein thrombosis
Hb: Hemoglobin
IL-6: Interleukin 6
NLR: Neutrophil to lymphocyte ratio
OR: Odd ratio
PE: Pulmonary embolism
PLR: Platelet to lymphocyte ratio
Post-op: Postoperative
Pre-op: Preoperative
RBC: Red blood cell
ROC: Receiver-operating characteristic
THA: Total hip arthroplasty
TJA: Total joint arthroplasty
TKA: Total knee arthroplasty
